# Ferulic Acid and Eugenol Have Different Abilities to Maintain Their Inhibitory Activity Against *Listeria monocytogenes* in Emulsified Systems

**DOI:** 10.3389/fmicb.2019.00137

**Published:** 2019-02-06

**Authors:** Aurélia Pernin, Véronique Bosc, Marie-Noëlle Maillard, Florence Dubois-Brissonnet

**Affiliations:** ^1^Micalis Institute, INRA, AgroParisTech, Université Paris-Saclay, Jouy-en-Josas, France; ^2^Ingénierie Procédés Aliments, AgroParisTech, INRA, Université Paris-Saclay, Massy, France

**Keywords:** phenolic compound, antimicrobial, growth inhibition, emulsion, partition coefficient, lipid droplets, phytophenol, foodborne pathogen

## Abstract

Natural phenolic compounds are found in large quantities in plants and plant extracts and byproducts from agro-industries. They could be used to ensure food quality and safety due to their antimicrobial properties demonstrated in systems such as culture media. The aim of this study was to evaluate the ability of two natural phenolic compounds, ferulic acid and eugenol, to maintain their inhibitory activity against the growth of *Listeria monocytogenes* in an oil-in-water emulsion, simulating a complex food system. The minimum inhibitory concentration (MIC) of each phenolic compound was first determined in culture medium, consisting of TS broth and an added emulsifier. Whey proteins and Tween 80 increased the MIC of the antimicrobial activity of eugenol. The MIC of ferulic acid was less affected by the addition of Tween 80. The inhibitory activities of both phenolic compounds were then compared at the same concentration in emulsions and their corresponding aqueous phases by following the growth of *L. monocytogenes* by plate counting. In emulsified systems, eugenol lost the high inhibitory activity observed in the aqueous phase, whereas ferulic acid retained it. The partition coefficient (logP_oct/wat_) appears to be a key factor. Eugenol (logP_oct/wat_ = 2.61) dispersed in the aqueous phase intercalates into the bacterial membrane and has high antimicrobial activity. In contrast, it likely preferentially partitions into the lipid droplets when dispersed in an emulsion, consequently losing its antimicrobial activity. As ferulic acid is more hydrophilic, a higher proportion probably remains in the aqueous phase of the emulsion, retaining its antimicrobial activity.

## Introduction

Natural phenolic compounds appear to be good candidates for ensuring the quality and safety of several perishable products, as they have been shown both antioxidant and antimicrobial activities (Brewer, 2011; Gyawali and Ibrahim, 2014; Pernin et al., 2018) and can be easily obtained in large quantities from plant extracts or byproducts from agro-industries (Balasundram et al., 2006; Tornuk et al., 2011). Many studies have already described the antimicrobial activity of phenolic compounds in culture media (Burt, 2004; Daglia, 2012) and there is an increasing interest to evaluate this activity in real foods or cosmetics. In contrast to simple culture media, these complex systems are highly heterogeneous and several regions with different physical properties can coexist. The effectiveness of antimicrobials in these complex systems is generally lower due to their interactions with matrix components and storage conditions (Weiss et al., 2015).

**FIGURE 1:**
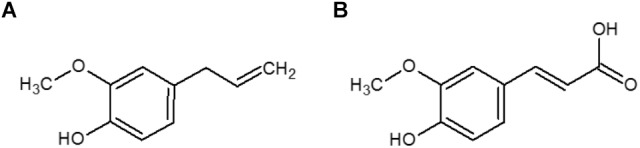
Chemical structures of eugenol **(A)** and ferulic acid **(B)**.

In a previous study, we evaluated the antimicrobial and antioxidant activities of a series of phenolic compounds in growth media. Ferulic acid and eugenol were identified among the few compounds to have both activities (Pernin et al., 2018). Ferulic acid is widely distributed in plants and can be found, for example, in sugar beet pulp and wheat or maize bran (Bonnin et al., 2002). Eugenol is mainly found in essential clove oil (Burt, 2004). These two molecules are known for their antimicrobial activities in culture media (Burt, 2004; Borges et al., 2013; Pernin et al., 2018, 2019) but only a few studies demonstrate their efficiencies in real food systems. Ferulic acid showed an inhibitory effect against *L. monocytogenes* in cheese (Takahashi et al., 2013; Van Tassell et al., 2015), smoked salmon (Takahashi et al., 2013), and salads (Takahashi et al., 2015b). Eugenol inhibited the growth of *L. monocytogenes* in cooked beef (Hao et al., 1998), in cabbage, barley and papaya pulp (Catherine et al., 2012). Ferulic acid and eugenol have similar chemical structures, both with a methoxyl group in the *ortho*-position of the phenolic group (Figure 1A,B), but ferulic acid possesses an acid function, contrary to eugenol. Moreover, these two compounds have different hydrophobic properties (logP_o/w_ = 2.61 for eugenol and logP_o/w_ = 1.67 and −1.81 for the undissociated and dissociated forms of ferulic acid respectively) that can affect their interactions with food components and distribution in heterogeneous systems.

The aim of this study was to evaluate the ability of these two compounds to inhibit the growth of *Listeria monocytogenes* in a realistic complex food system, *i.e.*, an oil-in-water emulsion that could mimic, for example, food sauces. We notably focused on the impact of the presence of two different emulsifiers (Tween 80 or whey proteins) and lipid droplets (fish oil) on the antimicrobial activity of these phenolic compounds. *L. monocytogenes* was chosen as the bacterial model because it is a ubiquitous Gram-positive pathogen that can potentially contaminate all stages of the food chain and is commonly found in ready-to-eat foods (Buchanan et al., 2017).

## Materials and Methods

### Bacterial Strain and Subculture Conditions

The strain used in this study was *Listeria monocytogenes* CNL 895805, serotype ½ a, isolated from sheep brain. It was graciously provided by P. Velge (INRA, Nouzilly) (Van Langendonck et al., 1998). Before each experiment, the strain, stored in cryovials at −80°C, was regrown in two successive subcultures in tryptic soy broth (TSB, Biomérieux, France) at 30°C.

### Phenolic Compounds and Other Chemicals

Eugenol was purchased from Jansen (Beerse, Belgium) and ferulic acid from Sigma-Aldrich (St. Quentin Fallavier, France) (Figure 1). The predicted partition coefficient (logP_o/w_) in octanol/water of eugenol is 2.61^1^. For ferulic acid, the logP_o/w_ of its undissociated and dissociated forms are respectively is 1.67 and −1.81^2^.

Hydrochloric acid (1 mol/L), sodium hydroxide (1 mol/L), and acetone were purchased from Carlo Erba (Fontenay-aux-Roses, France), Tween 80 (critical micellar concentration CMC = 19 mg/mL; Mahmood and Al-Koofee, 2013) from VWR (Fontenay-sous-Bois, France), and whey proteins (ProLacta 95) from Lactalis (Laval, France). Fish (tuna) oil (Omegavie^®^ 5/25 TG flavourless Qualitysilver^®^) was purchased from Polaris (Quimper, France) and stripped of its antioxidants before use according to a protocol adapted from Roman et al. (2013).

### Aqueous Phases for Emulsion Preparation

Aqueous phases consisted of TSB pH 7.2 with added emulsifiers and/or phenolic compounds, when appropriate. When necessary, the pH was adjusted to 5.5 with hydrochloric acid (1 mol/L) using a pH-meter (SI Analytics lab 870, Mainz, Germany). Tween 80 (7.1 g/L, *i.e.*, 0.5% (w/w) emulsion) was added to the TSB before pH adjustment and autoclave sterilization. Whey proteins (14.3 g/L, *i.e.*, 1% (w/w) emulsion) were added to TSB after autoclave sterilization and the mixture filtered through 0.22 μm filters (Stericup^®^ and Steritop^®^, Merck Millipore, Massachusetts).

The phenolic compounds were first prepared as stock solutions in acetone, due to their low solubility in TSB, and added at the appropriate concentration to the sterile aqueous phase. The maximum concentrations of phenolic compounds solubilized in acetone were 0.57 mol/L for ferulic acid and 1.68 mol/L for eugenol. The acetone was then evaporated under nitrogen flow. Bacterial growth controls were carried out to ensure the absence of inhibitory activity of acetone traces after evaporation.

### Determination of the Minimum Inhibitory Concentration of Ferulic Acid and Eugenol in Aqueous Phases

Inhibitory concentrations of eugenol and ferulic acid were determined using a method previously described with minor modifications (Guillier et al., 2007; Pernin et al., 2018). Bacterial growth was followed in an automatic spectrophotometer (Bioscreen C, Labsystems, Helsinki, Finland) by measuring the optical density (OD) at 600 nm for 72 h in two 100-well microplates.

A wide range of concentrations of eugenol and ferulic acid was tested to determine their inhibitory concentrations in TSB with each emulsifier. The inhibitory activity of eugenol was determined at pH 7.2 in the presence of Tween 80 (TSB-T80_pH7.2), whey proteins (TSB-WP_pH7.2), or without emulsifier as a control (TSB_pH7.2). The inhibitory activity of ferulic acid was determined at pH 7.2 without any emulsifier (TSB_pH7.2), at pH 5.5 in the presence of Tween 80 (TSB-T80_pH5.5), and at pH 5.5 without emulsifier (TSB_pH5.5).

The final concentrations of the phenolic compounds varied for eugenol from 0 to 8 mmol/L in TSB_pH7.2, 0–16 mmol/L in TSB-T80_pH7.2, and 0–12 mmol/L in TSB-WP_pH7.2 and for ferulic acid, from 0 to 30 mmol/L in TSB_pH7.2 and TSB_pH5.5 and from 0 to 12 mmol/L in TSB-T80_pH5.5. In the case of ferulic acid, the pH was adjusted to 5.5 or 7.2 using sodium hydroxide (1 mol/L). The concentrations were increased until total growth inhibition or they reached the solubility threshold. A total of 20–75 concentrations, prepared from at least two different solutions, were tested per compound in a given medium.

Two hundred microliters of the aqueous phases, with or without various concentrations of phenolic compounds, were added to each well. Each well was inoculated with a standardized inoculum at 1% (v/v from the second subculture (approximately 10^6^ CFU/mL)) and the microplates incubated at 30°C with slow and continuous shaking. At least two growth curves were acquired for each concentration. Antimicrobial activity is characterized by the minimum inhibitory concentration (MIC) and non-inhibitory concentration (NIC). The MIC is the concentration at which no bacterial growth is recorded and the NIC that below which the compound has no inhibitory activity. These values are expressed in mmol/L: the lower the MIC, the stronger the antimicrobial effect. These values were obtained after two modeling steps. First, the maximum specific growth rates (μ_max_) were estimated from the growth kinetics by fitting the modified Gompertz model (Guillier et al., 2007). Second, the MIC and NIC were determined for each phenolic compound by plotting growth rates transformed by square-root as a function of the concentration and modeling μ_max_ with the Lambert and Pearson model (Lambert and Pearson, 2000). The solver of Microsoft Excel^®^ (2013) was used to minimize the sum of squares and allowed estimation of the model parameters. The standard deviations (SD) of model parameters and sums of squares were calculated with SolverAid, a complementary macro (de Levie, 2012).

### Preparation of Emulsions

Emulsions were formulated with 30% (w/w) fish oil dispersed in the aqueous phase (see section Aqueous Phases for Emulsion Preparation). Fish oil was chosen for emulsion preparation because it contains a high proportion of long-chain unsaturated fatty acids from the omega-3 family, which are increasingly used in food products due to nutritional recommendations (FAO, 2010; Inra and Anses, 2013). Thirty-nine mL (*i.e.*, 36 g) of freshly stripped fish oil was added to 84 mL of the aqueous phase. The mixture was placed in a crystallizer filled with ice and water and emulsified using a T25 Ultra-Turrax homogenizer (Janke and Kunkel Ika Labortchnik, Staufen, Germany) with a S25KR-25F rotor-stator generator (previously autoclaved) at 9,500 rpm for 5 min, followed by 20 min with a sonicating probe (Sonifier^®^ Cell disrupter B15, Branson, Germany) at maximal magnitude with alternating cycles (10 s sonication/10 s rest). The particle size distributions of the emulsions were measured by laser light scattering (Mastersizer 2000, Malvern Instruments Ltd., Malvern, England). A refractive index ratio of 1.47 for the oil phase was used to calculate particle size distributions. The volume median diameter d(v,0.5) was determined: 1.24 ± 0.04 μm, which was stable throughout the duration of bacterial growth. All the systems displayed a mono-modal intensity-diameter distribution.

The antimicrobial activity of eugenol was tested in emulsions in the presence of two different emulsifiers, Tween 80 or whey proteins (Table 1) at the native pH of TSB (pH 7.2), which is close to that for the optimal growth of *L. monocytogenes* (Anses, 2011). The antimicrobial activity of ferulic acid was tested in emulsions in the presence of Tween 80 as emulsifier at pH 5.5, because ferulic acid is more active at acidic pH (Table 2). The antimicrobial activity of ferulic acid was not tested in emulsions containing whey proteins, as they precipitate at pH 5.5. The concentrations of phenolic compounds tested in the emulsions were chosen to be just above the MIC in the corresponding aqueous phases (or at the solubility threshold if the MIC was not reached, Table 2): 16 mmol/L eugenol in an emulsion with Tween 80 (Em-T80_pH7.2_Eu), 10 mmol/L eugenol in an emulsion with whey proteins (Em-WP_pH7.2_Eu), and 5.5 mmol/L ferulic acid in an emulsion with Tween 80 (Em-T80_pH5.5_Fe). The growth kinetics of *L. monocytogenes* in the emulsions with eugenol or ferulic acid was compared to respective control emulsions without any antimicrobial (Em-T80_pH7.2_Co, Em-WP_pH7.2_Co, and Em-T80_pH5.5_Co).

**Table 1:** Overview of the emulsified systems used in this study and corresponding aqueous phases.

Ingredient/Condition	TSB	pH		Whey proteins	Tween 80	Eugenol		Ferulic acid	Stripped fish oil
Value/Quantity (per L of aqueous phase)	30 g/L	7.2	5.5	14.3 g/L	7.1 g/L	16 mmol/L	10 mmol/L	5.5 mmol/L	30 % (w/w)
TSB-T80_pH7.2_Co	✓	✓			✓				
Em-T80_pH7.2_Co	✓	✓			✓				✓
TSB-T80_pH7.2_Eu	✓	✓			✓	✓			
Em-T80_pH7.2_Eu	✓	✓			✓	✓			✓
TSB-WP_pH7.2_Co	✓	✓		✓					
Em-WP_pH7.2_Co	✓	✓		✓					✓
TSB-WP_pH7.2_Eu	✓	✓		✓			✓		
Em-WP_pH7.2_Eu	✓	✓		✓			✓		✓
TSB-T80_pH5.5_Co	✓		✓		✓				
Em-T80_pH5.5_Co	✓		✓		✓				✓
TSB-T80_pH5.5_Fe	✓		✓		✓			✓	
Em-T80_pH5.5_Fe	✓		✓		✓			✓	✓

**Table 2:** *L. monocytogenes* growth rates (μ_max_), in different aqueous systems without phenolic compounds, determined using the Gompertz model and the non-inhibitory concentration (NIC) and minimum inhibitory concentration (MIC) of eugenol and ferulic acid, determined using the Lambert and Pearson model.

Phenolic compound	Aqueous systems	μ_max_ (h^−1^)	NIC (mmol/L)	MIC (mmol/L)
Eugenol	TSB_pH7.2	1.11 ± 0.11^A^	2.88 ± 0.20	5.62 ± 0.13
	TSB-WP_pH7.2	1.19 ± 0.02^A^	2.16 ± 0.22	7.95 ± 0.24
	TSB-T80_pH7.2	1.13 ± 0.02^A^	4.70 ± 0.59	>16^∗^
Ferulic acid	TSB_pH7.2	1.11 ± 0.11^A^	6.76 ± 0.85	>30^∗∗^
	TSB_pH5.5	0.91 ± 0.07 ^B^	0.33 ± 0.08	4.05 ± 0.26
	TSB-T80_pH5.5	0.86 ± 0.07 ^B^	0.54 ± 0.09	4.93 ± 0.17

*^∗^Solubility threshold of eugenol in TSB-Tween80. ^∗∗^Solubility threshold of ferulic acid in TSB pH7.2. For μ_max_, values are expressed in h^−1^ [mean ± standard deviation (n ≥ 3)] and different letters correspond to significantly different values according to the Newman–Keuls multiple comparison test (confidence interval 95%).*

### Bacterial Kinetics in Emulsions and Corresponding Aqueous Phases

Each emulsified system and its corresponding aqueous phase (Table 1) was inoculated with a standardized inoculum to obtain approximately 10^−1^ bacteria/mL (1% v/v, serially diluted from the second subculture). The antimicrobial concentrations were the same in the emulsions and their corresponding aqueous phases.

The flasks were incubated with stirring at 350 rpm on magnetic plates (MIXdrive 6HT, 2mag, Germany) at 25°C for a maximum of 72 h. At each sampling point, 1 mL (for aqueous phases) or 1.46 mL (for emulsions) was collected, serially diluted, and plated on tryptone soya agar (TSA, Biomérieux) using the drop plate method (Chen et al., 2003). Colony forming units (CFU) were enumerated after 24 h of incubation and the log_10_ CFU/mL of the aqueous phase was calculated. At least three independent repetitions were performed for each condition. The maximum specific growth rates (μ_max_) were estimated from the growth kinetics by fitting the modified Gompertz model (Guillier et al., 2007).

### Statistical Analysis

Growth rates (μ_max_) estimated without phenolic compounds and in emulsions and the corresponding aqueous phase came from experiments performed at least in triplicate. One-way analysis of variance (ANOVA) with a formulation effect was applied using XLStat 18.06 (Addinsoft, Paris, France). If significant effects were revealed (*p* < 0.05), an estimated mean for the growth rate was calculated and compared using the Newman-Keuls multiple comparison test (confidence interval 95%) to determine significant differences between formulations.

## Results

### MICs of Eugenol and Ferulic Acid in Aqueous Phases

**FIGURE 2:**
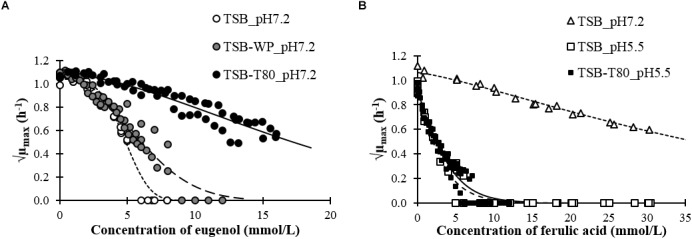
*L. monocytogenes* growth rates as a function of the concentrations of eugenol **(A)** or ferulic acid **(B)** in different aqueous systems (data fitted with the Lambert and Pearson model).

The growth rate of *L. monocytogenes* was similar in the absence or presence of emulsifier without the phenolic compounds at pH 7.2 or 5.5 (Table 2). We plotted the growth rates of *L. monocytogenes* in the aqueous phases as a function of the concentration of eugenol (Figure 2A) or ferulic acid (Figure 2B). The MIC and NIC of each compound in each aqueous phase are presented in Table 2.

The MIC of ferulic acid and eugenol in growth medium without emulsifier were in the same order of magnitude than MIC found in the literature against *L. monocytogenes* or other Gram positive bacteria by microdilution assays (Gutiérrez-Larraínzar et al., 2012; Borges et al., 2013; Van Tassell et al., 2015; Pernin et al., 2018, 2019) or by agar dilution assays (Takahashi et al., 2013).

The presence of emulsifier had a negative effect on the antimicrobial activity of eugenol. The MIC increased by 41% in the presence of whey proteins and more than 184% in the presence of Tween 80 (Table 2). The presence of Tween 80 also led to an increase in the NIC of eugenol.

The MIC was not attained before the solubility threshold (30 mmol/L) of ferulic acid at pH 7.2. Nevertheless, ferulic acid inhibited the growth of *L. monocytogenes* at pH 7.2, as it decreased from 1.1 h^−1^ (without ferulic acid) to 0.4 h^−1^ at 30 mmol/L (√0.4 = 0.6 h^−1/2^ in Figure 2). The antimicrobial activity of ferulic acid at pH 5.5 was much higher than at pH 7.2. The presence of Tween 80 had a small negative effect on the inhibitory activity of ferulic acid with a 22% increase of the MIC. The effect of whey proteins could not be tested at pH 5.5 because of protein aggregation.

### Inhibitory Activity of Eugenol and Ferulic Acid in Emulsions and Their Corresponding Aqueous Phases

The presence of oil and emulsifiers alone at pH 7.2 did not significantly affect the growth rate of *L. monocytogenes* (Table 3, 0.97 ± 0.11 for emulsified systems with Tween 80 *vs.* 1.21 ± 0.16 h^−1^ for the corresponding aqueous phase and 1.05 ± 0.14 for emulsified systems with whey proteins *vs.* 1.22 ± 0.08 h^−1^ the corresponding aqueous phase). In contrast, the presence of oil and Tween 80 at pH 5.5 significantly decreased the growth rate of *L. monocytogenes* (Table 3, 0.62 ± 0.08 *vs.* 0.96 ± 0.11 h^−1^).

**Table 3:** *L. monocytogenes* growth rates (μ_max_) determined using the Gompertz model in different aqueous and emulsified systems, with or without phenolic compounds.

	T80_pH7.2		WP_pH7.2		T80_pH5.5	
	Control	Eugenol (16 mmol/L)	Control	Eugenol (10 mmol/L)	Control	Ferulic acid (5.5 mmol/L)
Aqueous phases	1.21 ± 0.16 ^A^	0.39 ± 0.07 ^C^	1.22 ± 0.08 ^A^	0.00 ± 0.00 ^D^	0.96 ± 0.11 ^A^	0.00 ± 0.00 ^D^
Emulsified systems	0.97 ± 0.11 ^A^	1.13 ± 0.14 ^A^	1.05 ± 0.14 ^A^	1.25 ± 0.13 ^A^	0.62 ± 0.08 ^B^	0.00 ± 0.00 ^D^

*Values are expressed in h^−1^ [mean ± standard deviation (n ≥ 3)] and different letters correspond to significantly different values according to the Newman–Keuls multiple comparison test (confidence interval 95%).*

**FIGURE 3:**
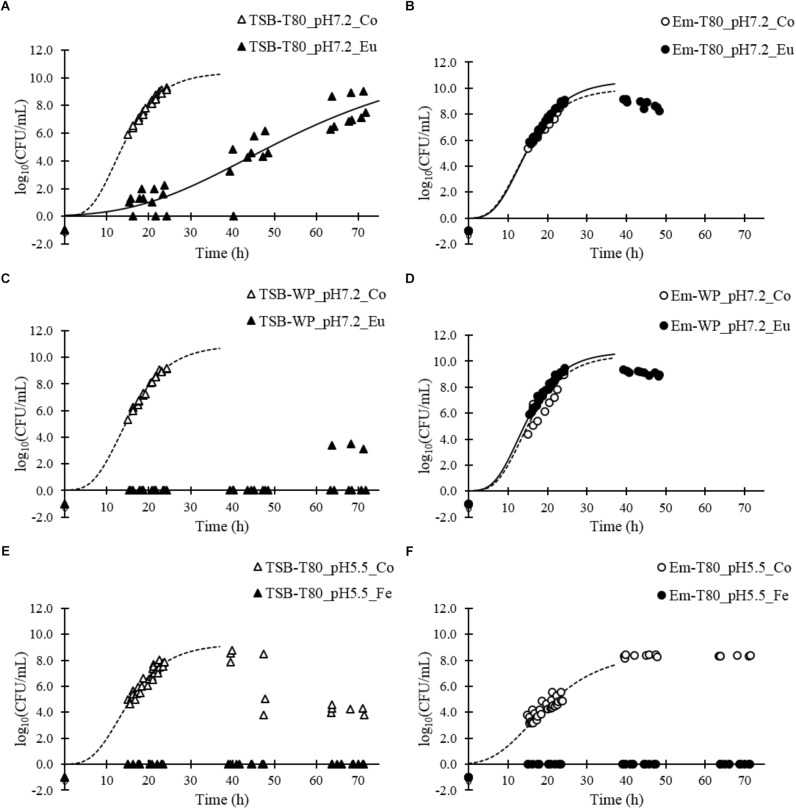
Growth curves of *L. monocytogenes* in different aqueous and emulsified systems in the presence of eugenol or ferulic acid: aqueous system **(A)** and emulsion **(B)** formulated with Tween 80 at pH 7.2 with or without eugenol (16 mmol/L); aqueous system **(C)** and emulsion **(D)** formulated with whey proteins at pH 7.2 with or without eugenol (10 mmol/L); aqueous system **(E)** and emulsion **(F)** formulated with Tween 80 at pH 5.5 with or without ferulic acid (5.5 mmol/L). Growth curves were fitted using the Gompertz model until 40 h (until the bacterial counts decreased).

Eugenol at a concentration of 16 mmol/L in TSB-Tween 80 at pH 7.2 demonstrated incomplete, but significant, inhibitory activity: the growth rate of *L. monocytogenes* was 0.39 ± 0.07 h^−1^ *vs.* 1.21 ± 0.16 h^−1^ for the control (Figure 3A and Table 3). In contrast, eugenol at the same concentration did not show any inhibitory activity in an emulsion with Tween 80 at pH 7.2: the bacterial growth rate was 1.13 ± 0.14 h^−1^ *vs.* 0.97 ± 0.11 h^−1^ for the control (Figure 3B and Table 3).

We obtained similar results for 10 mmol/L eugenol in emulsions with whey proteins at pH 7.2. The inhibition of bacterial growth was complete in TSB-WP-Eu, whereas the bacterial growth rate was 1.22 ± 0.08 h^−1^ without eugenol (Figure 3C and Table 3). However, eugenol completely lost its inhibitory activity when in an emulsion (Figure 3D and Table 3).

Ferulic acid, at a concentration of 5.5 mmol/L, completely inhibited the growth of *L. monocytogenes* in TSB-Tween 80 at pH 5.5 (Figure 3E and Table 3), as well as in the corresponding emulsion (Figure 3F and Table 3).

Overall, the results show that the presence of oil and emulsifiers alter the inhibitory activity of eugenol but not that of ferulic acid.

## Discussion

### Effect of Emulsifiers on Inhibitory Activity of Phenolic Compounds in Aqueous Phases

The presence of either emulsifier resulted in an increase in the MICs of eugenol and ferulic acid, whereas it had no significant effect on the growth rate of *L. monocytogenes* in the absence of the antimicrobials (Table 2). Similarly, several studies have shown that the presence of emulsifiers can prevent essential oils and/or their pure compounds (generally eugenol, thymol, or carvacrol) from interacting with microorganisms (Juven et al., 1994; Hammer et al., 1999; Hammer and Carson, 2011).

Juven et al. (1994) showed that the addition of Tween 80 in the culture medium decreases the antimicrobial activity of phenolic compounds. Moreover, they showed that the higher the concentration of Tween 80 (1,000 μL/L *vs.* 125 μL/L), the higher the loss of activity of 140 mg/mL of thymol against *Salmonella* Typhimurium in nutrient agar (10^9.1^ CFU/mL *vs.* < 10^1^ CFU/mL recovery) (Juven et al., 1994). Several possibilities can explain this loss of activity. First, Tween 80 may form a protective coating around the bacteria that could prevent phenolic compounds from accessing the bacterial membrane. However, it is more likely that the emulsifier interacts with the phenolic compounds, leading to a decrease in their availability to contact the bacteria. Indeed, Tween 80 molecules are low-molecular weight emulsifiers composed of a polar polyethoxy-head and a hydrophobic tail consisting of oleic acid. They are known to form micelles when present above their critical micellar concentration (CMC) in aqueous systems (Berton-Carabin et al., 2014), which was the case in our study. Tween 80 was used at a concentration of 7.1 g/L, approximately 500 times higher than the CMC (19 mg/mL). These micelles form specific hydrophobic environments in which other molecules can be solubilized (Berton-Carabin et al., 2014). Eugenol and ferulic acid molecules could be trapped in micelles, thus being unavailable to interact with bacteria. As a consequence, higher concentrations are needed to obtain the same level of bacterial growth inhibition. The difference observed between eugenol and ferulic acid is probably due to the respective hydrophobicity (octanol/water partition coefficients, logP_o/w_) of these two molecules. Eugenol, with a logP_o/w_ of 2.61, is likely more easily partitioned into Tween 80 micelles than ferulic acid (logP_o/w_ = 1.67 for the undissociated form).

The presence of whey proteins in the TSB also led to a slight increase in the MIC of eugenol. Proteins were previously shown to interfere with antimicrobial activity. For example, Juven et al. (1994) showed that the addition of 9 g/L bovine serum albumin (BSA) to nutrient agar suppresses the inhibitory capacity of thymol against *Salmonella* Typhimurium. This could be explained by the well-known ability of proteins to bind phenolic compounds through van der Waals interactions (Weiss et al., 2015) and thus inactivate them, decreasing the number of molecules available for inhibiting bacterial growth. Indeed, Bouarab-Chibane et al. (2018) showed that the decrease of antimicrobial activity of phenolic compounds with a molecular weight close to our compounds (resveratrol, naphthazarin, and chrysin) directly correlated with their higher affinity for bovine meat proteins, determined through the measurements of partition coefficients of phenolic compounds between a 20% (w/w) bovine meat protein suspension and its ultrafiltrate without proteins. Moreover, Reiners et al. (2000) used affinity chromatography at pH 3.0 to show the ability of eugenol to bind to β-lactoglobulin, the main protein from whey proteins. However, interactions between protein and phenolic compounds are complex and depend on several parameters, such as pH, temperature, protein type and concentration, and the type and structure of the phenolic compounds (Ozdal et al., 2013). Bouarab-Chibane et al. (2018) also showed that the antimicrobial activity of phenolic compounds in the presence of proteins is better preserved at low temperatures, since hydrogen bonds between phenolic compounds and the bacterial surface are favored at the expense of van der Waals and hydrophobic interactions between the phenolic compounds and proteins.

In contrast, some studies have reported a positive impact of some emulsifiers, such as monolaurin (Blaszyk and Holley, 1998), surfynol (Gaysinsky et al., 2005a,b), soluble soy bean polysaccharides (Wu et al., 2014), and rhamnolipids (Haba et al., 2014), on the antimicrobial activity of essential oils and/or their pure constitutive compounds. This may be due to the ability of these emulsifiers to disperse in the aqueous phase, thus preventing their binding to the hydrophobic phenolic compounds (Blaszyk and Holley, 1998; Gaysinsky et al., 2005a,b). Another possibility suggested in the literature is that the emulsifiers cited above may enhance the efficiency of antimicrobials by increasing their interaction with the bacterial membrane (Donsì and Ferrari, 2016). In this case, interactions between phenolic compounds and bacterial membranes are probably tighter than those between emulsifiers and phenolic compounds. However, the large differences between these emulsifiers and those used in our study, in terms of chemical structures, charges, size, and physical properties, could explain the difference of behavior.

### Effect of the Presence of Oil Droplets on the Inhibitory Activity of Phenolic Compounds

Eugenol, in contrast to ferulic acid, completely lost its inhibitory activity when added to emulsified systems, whereas they were both efficient in the corresponding aqueous phases containing emulsifiers. To our knowledge, the antimicrobial activity of ferulic acid in emulsions has never been studied, but some data are available in the literature concerning eugenol and other simple phenols from essential oils (Sznitowska et al., 2002; Han and Washington, 2005; Gaysinsky et al., 2007; Gutierrez et al., 2008; Rattanachaikunsopon and Phumkhachorn, 2010; Cava-Roda et al., 2012; Chang et al., 2012; Trinh Thi Thanh et al., 2013). Many studies have shown that the addition of oil to an aqueous phase has negative effects on antimicrobial activity. For example, Chang et al. (2012) showed that increasing the percentage of corn oil in emulsions from 6 to 9% (w/w) led to a 16-fold increase in the MIC of thyme essential oil against *Zygosaccharomyces bailii*. Similarly, sunflower oil added with soya lecithin to TSB (final concentration in the emulsion of 2.5%, w/w) increased the MIC of cinnamon essential oil (composed of 90% *trans*-cinnamaldehyde) and pure *trans* cinnamaldehyde by approximately fivefold on *Listeria innocua* (Trinh Thi Thanh et al., 2013). In contrast, several studies reported no loss of efficiency for some phenolic compounds extracted from essential oils in emulsions (Donsì et al., 2012; Terjung et al., 2012; Ghosh et al., 2014; Li et al., 2015). However, the antimicrobial concentrations in the emulsified systems were much higher (from 130 to 260 mmol/L) than in our study.

**FIGURE 4:**
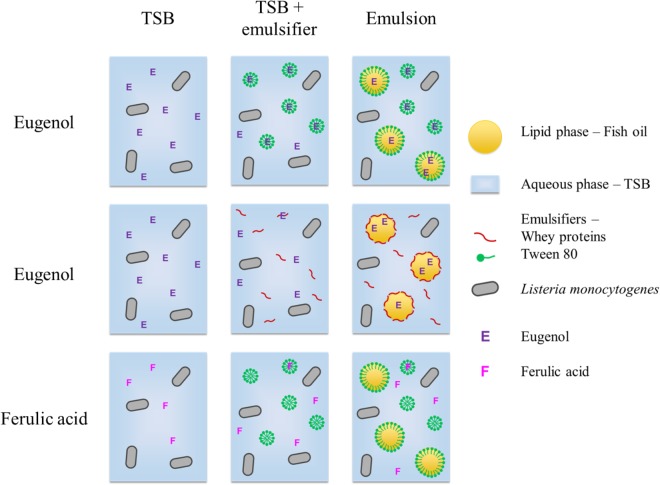
Hypothesis for the partition of eugenol and ferulic acid in the different phases in aqueous and emulsified systems.

The loss of antimicrobial activity in emulsions is generally believed to be due to the preferential localization of the compounds to specific regions of the highly structured systems. The presence of a lipid phase can notably affect the distribution of components based on their affinity for hydrophobic or hydrophilic environments. The antimicrobial concentration available in the aqueous phase, in which the microorganisms are located, depends on the repartition of the compound between phases (Donsì et al., 2012; Terjung et al., 2012; Donsì and Ferrari, 2016). The difference observed in this study between eugenol and ferulic acid is likely due to their difference in hydrophobicity. Indeed, based on its logP_o/w_ (2.61), eugenol should more easily partition into oil droplets than ferulic acid (logP_o/w_ of undissociated and dissociated forms are 1.67 and −1.81 respectively). Thus, these two compounds differentially partition into the aqueous phases and emulsions based on their hydrophobic character (Figure 4): the more hydrophobic the compound, the more inefficient it will be in the presence of lipid droplets. Between the two forms of ferulic acid, the dissociated form is the one that remains preferentially in aqueous phase, because of its lower logP_o/w_. It can be noted that the dissociated form of ferulic acid, unlike that of most phenolic acids, was recently shown to have a significant inhibitory efficiency against *L. monocytogenes* (Pernin et al., 2019).

Paradoxically, characteristic hydrophobicity parameters, such as octanol/water partition coefficients, are often used to predict the efficiency of membrane-active compounds, such as simple phenols: the more hydrophobic, the more they can partition into double phospholipid layers (Ultee et al., 2002; Burt, 2004). In this context, compound selection, such as antimicrobials in emulsions, may face a contradiction. In aqueous phases, a phenolic compound with a high logP, such as eugenol, would preferentially partition into the bacterial membrane, thus exhibiting strong antimicrobial activity. In contrast, when dispersed in an emulsion, the same compound would preferentially migrate into the lipid droplets. Consequently, its concentration in the aqueous phase would be dramatically lower, along with its antimicrobial activity. This is probably what happened in previous studies about emulsions with thyme essential oil, composed of carvacrol and thymol (logP_o/w_ 3.43) (Chang et al., 2012), and with cinnamon essential oil, composed of *trans* cinnamaldehyde (logP_o/w_ 1.98) (Trinh Thi Thanh et al., 2013). In contrast, a less hydrophobic compound, such as ferulic acid, probably remains in higher proportion in the aqueous phase of the emulsion, therefore retaining their antimicrobial activity. Thus, in order to optimize antimicrobial activity in complex emulsified systems such as foods or cosmetic matrices, it is better to choose a compound such as ferulic acid that has a multifactorial mode of action and that is not excessively hydrophobic for maintaining a sufficient proportion in the aqueous phase. Ferulic acid is especially interesting since *L. monocytogenes* does not develop tolerance after exposure to low concentrations (Takahashi et al., 2015a).

## Conclusion

We demonstrate that emulsifiers, such as whey proteins and Tween 80 have a negative impact on the antimicrobial activity of eugenol. The MIC of ferulic acid was less affected by the addition of Tween 80. This effect appears to be mainly due to potential interactions between phenolic compounds and emulsifiers. Moreover, eugenol is not an attractive phenolic antimicrobial in emulsified systems, as opposed to ferulic acid. This appears to be mainly due to the presence of oil, that likely traps eugenol in the lipid phase. A much higher concentration of eugenol would probably be needed to inhibit bacterial growth in such emulsions, but it would negatively affect their organoleptic properties. In contrast, ferulic acid at pH 5.5 is an efficient antimicrobial at low concentrations in such systems, probably because it is less hydrophobic. These results highlight the necessity to select natural preservatives after testing them in complex media, such as lipid-rich systems close to realistic conditions for food or cosmetic applications.

## Author Contributions

AP, M-NM, and FD-B designed the study. AP, M-NM, and VB designed the emulsified system. AP carried out most of the experiments. AP and FD-B analyzed and interpreted the data. AP and FD-B wrote the manuscript with valuable feedback from M-NM and VB.

## Conflict of Interest Statement

The authors declare that the research was conducted in the absence of any commercial or financial relationships that could be construed as a potential conflict of interest.

## Footnotes

1 https://chemicalize.com/

2 https://chemicalize.com/

## Abbreviations

MIC: minimum inhibitory concentration
NIC: non-inhibitory concentration.
